# Transgenic human C-reactive protein affects oxidative stress but not inflammation biomarkers in the aorta of spontaneously hypertensive rats

**DOI:** 10.1186/s12872-024-03870-7

**Published:** 2024-04-16

**Authors:** Ivana Nemeckova, Samira Eissazadeh, Jana Urbankova Rathouska, Jan Silhavy, Hana Malinska, Michal Pravenec, Petr Nachtigal

**Affiliations:** 1 Department of Biological and Medical Sciences, Faculty of Pharmacy in Hradec Kralove, Charles University, Heyrovskeho 1203, Hradec Kralove, 500 05 Czech Republic; 2 Institute of Physiology, Academy of Sciences of the Czech Republic, Prague, Czech Republic; 3 Center for Experimental Medicine, Institute for Clinical and Experimental Medicine, Prague, Czech Republic

**Keywords:** C-reactive protein, Spontaneously hypertensive rat, Aorta, Endothelial dysfunction, Oxidative stress

## Abstract

**Background:**

C-reactive protein (CRP) is an acute inflammatory protein detected in obese patients with metabolic syndrome. Moreover, increased CRP levels have been linked with atherosclerotic disease, congestive heart failure, and ischemic heart disease, suggesting that it is not only a biomarker but also plays an active role in the pathophysiology of cardiovascular diseases. Since endothelial dysfunction plays an essential role in various cardiovascular pathologies and is characterized by increased expression of cell adhesion molecules and inflammatory markers, we aimed to detect specific markers of endothelial dysfunction, inflammation, and oxidative stress in spontaneously hypertensive rats (SHR) expressing human CRP. This model is genetically predisposed to the development of the metabolic syndrome.

**Methods:**

Transgenic SHR male rats (SHR-CRP) and non-transgenic SHR (SHR) at the age of 8 months were used. Metabolic profile (including serum and tissue triglyceride (TAG), serum insulin concentrations, insulin-stimulated incorporation of glucose, and serum non-esterified fatty acids (NEFA) levels) was measured. In addition, human serum CRP, MCP-1 (monocyte chemoattractant protein-1), and adiponectin were evaluated by means of ELISA, histological analysis was used to study morphological changes in the aorta, and western blot analysis of aortic tissue was performed to detect expression of endothelial, inflammatory, and oxidative stress markers.

**Results:**

The presence of human CRP was associated with significantly decreased insulin-stimulated glycogenesis in skeletal muscle, increased muscle and hepatic accumulation of TAG and decreased plasmatic cGMP concentrations, reduced adiponectin levels, and increased monocyte chemoattractant protein-1 (MCP-1) levels in the blood, suggesting pro-inflammatory and presence of multiple features of metabolic syndrome in SHR-CRP animals. Histological analysis of aortic sections did not reveal any visible morphological changes in animals from both SHR and SHR-CRP rats. Western blot analysis of the expression of proteins related to the proper function of endothelium demonstrated significant differences in the expression of p-eNOS/eNOS in the aorta, although endoglin (ENG) protein expression remained unaffected. In addition, the presence of human CRP in SHR in this study did not affect the expression of inflammatory markers, namely p-NFkB, P-selectin, and COX2 in the aorta. On the other hand, biomarkers related to oxidative stress, such as HO-1 and SOD3, were significantly changed, indicating the induction of oxidative stress.

**Conclusions:**

Our findings demonstrate that CRP alone cannot fully induce the expression of endothelial dysfunction biomarkers, suggesting other risk factors of cardiovascular disorders are necessary to be involved to induce endothelial dysfunction with CRP.

**Supplementary Information:**

The online version contains supplementary material available at 10.1186/s12872-024-03870-7.

## Introduction

C-reactive protein (CRP) is a classical acute-phase protein, which is a highly sensitive systemic marker of inflammation and tissue damage [1]. Except for being a part of an immune reaction during infection or extensive tissue injury, several papers demonstrated the important role of CRP as a biomarker of various cardiovascular pathologies, including atherosclerosis and ischemic heart disease [2].

Interestingly, the precise role of CRP in the development of endothelial dysfunction and atherosclerosis, which are considered inflammatory diseases, has not yet been fully elucidated. From the morphological point of view, endothelial dysfunction is characterized by increased expression of cell adhesion molecules and inflammatory markers [3], reduced expression of endothelial NO-synthase (eNOS), and endoglin (ENG), a transmembrane glycoprotein also known as CD105 or TGF-β receptor III, which is predominantly expressed by endothelial cells [4]. Moreover, endothelial dysfunction is associated with excessive production of reactive oxygen species (ROS) and impaired antioxidant defenses characterized as oxidative stress [5].

There are some studies demonstrating the pro-atherogenic role of CRP in both in vitro and in vivo, suggesting effects on eNOS activity [6, 7], prothrombotic state, and foam cell formation [8]. In addition, the pro-inflammatory role of CRP has been confirmed in vitro by direct induction of human umbilical vein endothelial cells (HUVECs) to express monocyte chemoattractant protein 1 (MCP-1), which has a crucial role in recruiting monocytes into the vessel wall [5].

On the other hand, it is worth mentioning that a genetic study did not support the pro-atherogenic role of CRP [9]. It has been shown that high levels of CRP are associated with oxidative stress in patients without clinically relevant cardiovascular history [10]. Hemeoxygenase-1 (HO-1) plays a crucial role in vascular protection in inflammatory and oxidative stress processes. HO-1 is an inducible protein that is notably up-regulated, especially in endothelial cells, macrophages, foam cells, and vascular smooth muscle cells under various pathological stimuli [11]. It has significant antioxidant properties via the degradation of prooxidative heme and the production of protective molecules of biliverdin and bilirubin [12].

Moreover, it has been shown that induction of HO-1 contributes to the reduction of oxidative stress and inflammation in kidneys in spontaneously hypertensive rats (SHR). In addition to inducible proteins, constitutive enzymes such as superoxide dismutase (SOD) regulate redox homeostasis. There are 3 isoforms of SOD, and the most abundant isoform in the vascular wall is SOD3, which counts for almost half of the total SOD activity in the aorta [5]. SHR expressing human CRP was introduced in order to study the direct effects of CRP in various organs. Indeed, these rats were demonstrated to have symptoms of metabolic syndrome, including arterial hypertension, hyperinsulinemia, inflammation, and oxidative stress [13]. Moreover, CRP effects in this transgenic model are modulated by the genetic predisposition of SHR to the development of hypertension, where CRP can escalate future cardiovascular complications [14].

Interestingly, the possible involvement of CRP in the development of endothelial dysfunction in the aorta has not been studied in SHR-CRP rats so far. Indeed, several studies showed the development of aortic endothelial dysfunction in different rat models, suggesting the aorta is a suitable blood vessel for studying the development of endothelial dysfunction [15, 16]. Therefore, we hypothesized that CRP would increase the expression of endothelial dysfunction, inflammation, and oxidative stress biomarkers in SHR-expressing human CRP.

## Materials and methods

### Animals and study design

Eight months old transgenic SHR male rats (hereafter referred to as transgenic or SHR-CRP) (*n* = 5) were derived by microinjections of SHR ova with a previously described construct containing the cDNA for human CRP under control of the apoE promoter to drive expression of the CRP transgene in the liver producing CRP. In detail, fertilized eggs were microinjected with a DNA construct consisting of 1.13 kb human C-reactive protein (hCRP) cDNA under the control of liver-specific expression elements from the human apolipoprotein E gene with 4 copies of the chicken beta-globin insulator. Insulators can prevent the position effect of transgene CRP [13]. Non-transgenic male SHR (hereafter referred to as control or SHR) age-matched rats were used as a control (*n* = 5).

The rats were housed in an air-conditioned animal facility and allowed free access to a standard laboratory diet and water. At the end of the experiment, animals were sacrificed by cervical dislocation in a postprandial state, followed by the collecting of tissues and blood samples for further analysis. All experiments were performed using collected tissues only, not live animals. These experiments were performed in agreement with the Animal Protection Law of the Czech Republic and were approved by the Ethics Committee of the Institute of Physiology, Czech Academy of Sciences, Prague (Permit Number: 66/2014). The study was reported in accordance with ARRIVE guidelines.

### Biochemical and metabolic analyses

According to the manufacturer’s instructions, human serum CRP was measured using ELISA kit (Alpha Diagnostics International, TX, USA). Serum and tissue triglyceride (TAG) concentrations were measured by standard enzymatic methods (Pliva-Lachema, Czech Republic). To determine TAG content in tissues, samples were extracted in a chloroform/methanol mixture. Serum insulin concentrations were determined using a rat insulin ELISA kit (Mercodia, Sweden). Serum MCP-1 was determined using the rat ELISA kit from eBioscience, Bender MedSystems Biocenter, Austria. Serum adiponectin was determined by means of a rat ELISA kit (B-Bridge International, Inc., CA, USA). Insulin-stimulated incorporation of glucose into glycogen was determined in isolated soleus muscle. The soleus muscle was incubated for 2 h in Krebs-Ringer bicarbonate buffer (pH 7.4) gassed with 95% O_2_ and 5% CO_2_ that contained 5.5 mM unlabeled glucose, 0.5 μCi/ml of ^14^C-U glucose, and 3 mg/ml bovine serum albumin (Fraction V, Sigma, Czech Republic) with or without 250 μunits/ml insulin. After 2 h of incubation, glycogen was extracted, and glucose incorporation into glycogen was determined. Levels of plasma cyclic guanosine monophosphate (cGMP) were measured in acetylated supernatant using a radioimmunoassay kit (IBL International GmbH, Germany). Serum nonesterified fatty acids (NEFA) levels were determined using an acyl-CoA oxidase-based colorimetric kit (Roche Diagnostics GmbH, Germany).

### Histological analysis

Aorta samples were fixed in 4% formaldehyde, embedded into paraffine and 5 μm serial cross-sections were cut. Slides were stained with hematoxylin and eosin to study possible morphological changes. Microscopic pictures were captured by an Olympus AX 70 microscope with an incorporated Nikon DS-Fi3 high-definition color microscope camera and image analysis software NIS (Laboratory Imaging, Czech Republic).

### Western blot analysis

Samples of the aorta from all rats were homogenized in RIPA lysis buffer (Sigma-Aldrich, MA, USA) containing proteases (SERVA Electrophoresis, Germany) and phosphatases (Thermo Fisher Scientific Inc., IL, USA) inhibitors, as described previously [17]. Proteins were separated by sodium dodecyl sulfate-polyacrylamide gel electrophoresis (SDS-PAGE) and electrically transferred onto PVDF membrane (Millipore, NY, USA) using Trans-Blot SD Semi-Dry Electrophoretic Transfer Cell (Bio-Rad, CA, USA). The membranes were blocked for 1 h with 5% non-fat dry milk (Cell Signaling Technology, MA, USA) in Tris (Serva, OK, USA) buffer saline containing 0.1% Tween-20 (Sigma-Aldrich, MA, USA) at room temperature and then incubated with appropriate antibodies (Table 1) for 16 h at 4ºC. Horseradish peroxidase-conjugated secondary antibodies were from Sigma-Aldrich (MA, USA) or Abcam (UK), as described [17]. Membranes were developed using enhanced chemiluminescent reagents (Thermo Fisher Scientific Inc., IL, USA) and subsequently exposed to X-ray films (Foma, Czech Republic). Quantification of immunoreactive bands on the exposed films was performed by NIS imaging software (Laboratory Imaging, Czech Republic). The equal loading of proteins onto the gel was confirmed by immunodetection of glyceraldehyde-3-phosphate dehydrogenase (GAPDH).

**Table 1:** List of antibodies for western blot analysis

Antibody	Supply	Dilution	Secondary antibody dilution
eNOS	Santa Cruz Biotechnology, Inc., CA, USA	1:200	1:1000
p-eNOS (Ser1177)	Santa Cruz Biotechnology, Inc., CA, USA	1:100	1:2000
ENG	Santa Cruz Biotechnology, Inc., CA, USA	1:200	1:1000
p-NFkB	Santa Cruz Biotechnology, Inc., CA, USA	1:200	1:1000
P-selectin	Santa Cruz Biotechnology, Inc., CA, USA	1:200	1:1000
COX2	Proteintech, IL, USA	1:500	1:1000
HO-1	Abcam, Camb, UK	1:2000	1:4000
SOD3	Abcam, Camb, UK	1:1000	1:2000
GAPDH	Sigma-Aldrich, MA, USA	1:10000	1:20000

### Statistical analysis

The statistical analysis was performed by GraphPad Prism 9.2 software (GraphPad Software, Inc., CA, USA). All data are presented as median with interquartile range (IQR). Direct group-group comparisons were carried out using the non-parametric Mann-Whitney test. *P* values of 0.05 or less were considered statistically significant.

## Results

### Biochemical analysis of blood and metabolic profile in SHR and SHR-CRP rats

**Fig. 1:**
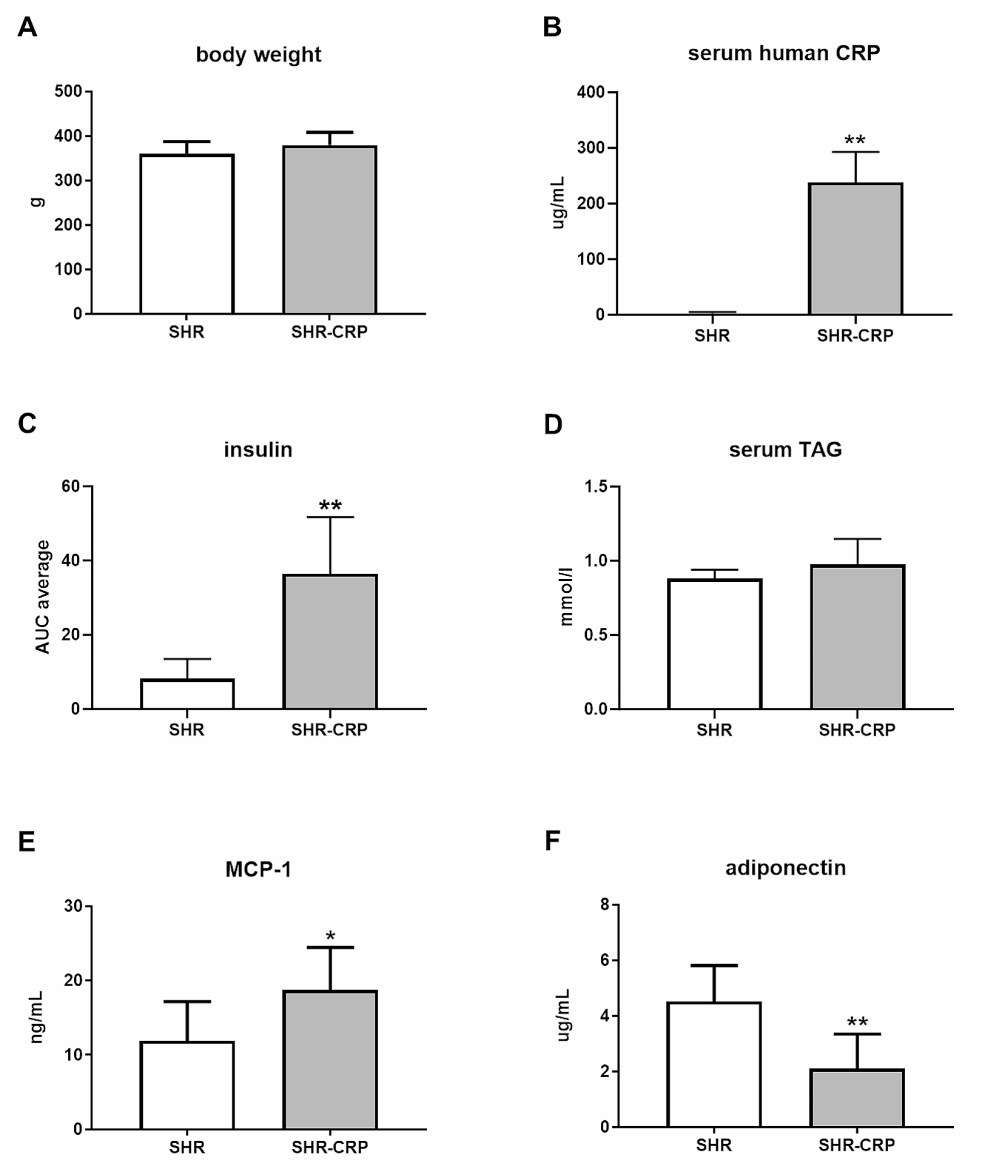
Body weight and biochemical serum analysis. Body weight (**A**), serum human CRP levels (**B**), insulin levels during OGTT (**C**), serum TAG levels (**D**), serum MCP-1 levels (**E**), and serum adiponectin levels (**F**) in control SHR and transgenic SHR-CRP animals. The area under the curve (AUC) for insulin was calculated over the 120 min period and presented as AUC average. Data are presented as median with IQR and Mann-Whitney test. *p ≤ 0.05, **p ≤ 0.01

Biochemical analysis of human CRP was performed in order to confirm the successful generation of transgenic animals. Body weight was similar in both groups (Fig. 1A). ELISA results showed significantly higher levels of human CRP in transgenic SHR-CRP rats when compared to SHR rats (Fig. 1B). Serum insulin levels were significantly increased in SHR-CRP rats during the oral glucose tolerance test (OGTT) (Fig. 1C), suggesting impaired insulin reactivity. However, serum TAG levels did not differ significantly when compared to SHR rats (Fig. 1D). Serum levels of pro-inflammatory MCP-1 were significantly increased in SHR-CRP rats (Fig. 1E). On the contrary, serum levels of adiponectin were significantly lower in SHR-CRP rats (Fig. 1F).

Moreover, transgenic overexpression of human CRP was associated with significantly decreased insulin-stimulated glycogenesis in skeletal muscle, increased muscle and hepatic accumulation of TAG, and decreased plasmatic cGMP concentrations, suggesting the presence of multiple features of metabolic syndrome in SHR-CRP animals. On the other hand, the weight and metabolic activity of visceral adipose tissue (epididymal and perirenal adipose tissue, NEFA) did not significantly differ between SHR and SHR-CRP groups (Table 2).

**Table 2:** Metabolic parameters in control SHR and transgenic SHR-CRP animals

	SHR	SHR-CRP
Insulin-stimulated glycogenesis	278 ± 32	174 ± 18*
(nmol glucose/mg prot/2 hours)		
Muscle triglycerides (umol/g)	1.84 ± 0.21	2.90 ± 0.29*
Hepatic triglycerides (umol/g)	5.83 ± 0.28	7.27 ± 0.64*
Plasma cGMP (pmol/l)	19.31 ± 3.94	9.31 ± 4.63*
Relative weight of epididymal adipose tissue (g/100 g bwt)	0.733 ± 0.041	0.637 ± 0.030
Relative weight of perirenal adipose tissue (g/100 g bwt)	0.342 ± 0.042	0.248 ± 0.043
NEFA (mmol/l)	0.639 ± 0.028	0.674 ± 0.051

cGMP, cyclic guanosine monophosphate; NEFA, non-esterified fatty acids. Data are presented as mean ± SEM, Mann-Whitney test. *p ≤ 0.05

### Histological analysis

**Fig. 2:**
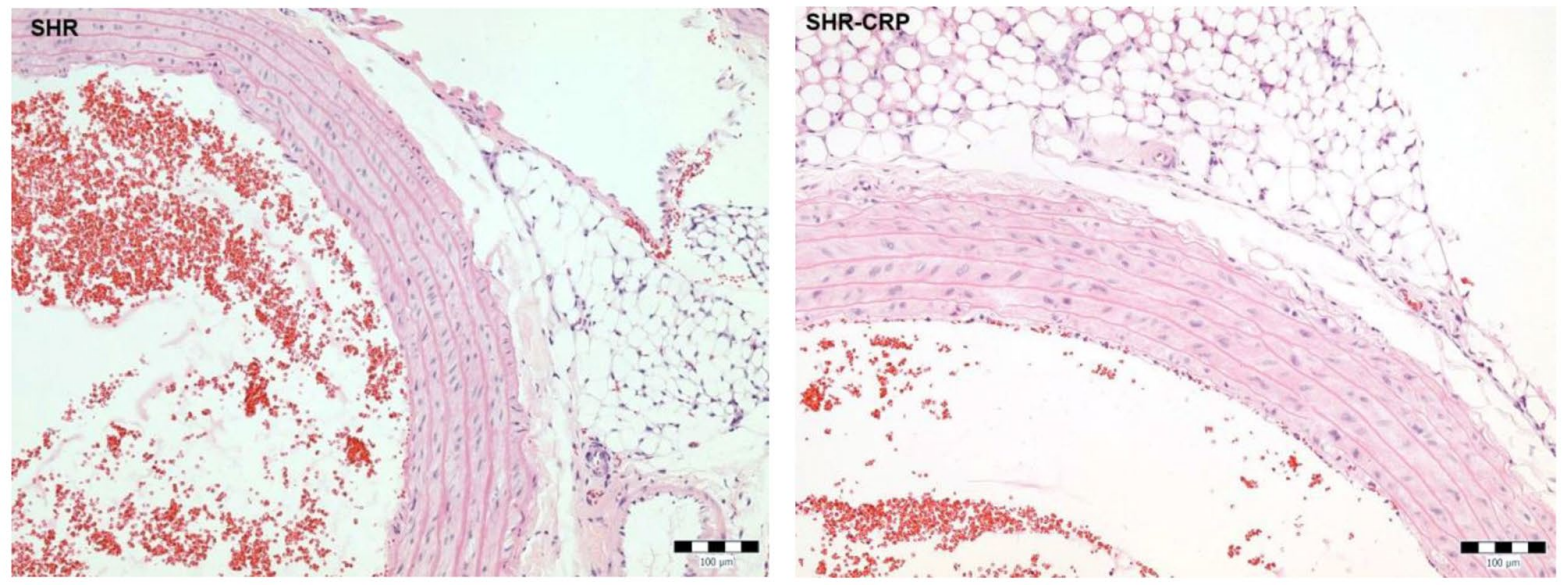
Histological evaluation of aorta. Representative photomicrographs of hematoxylin and eosin staining of the aortas from SHR and SHR-CRP animals. *Bar 100 μm*

Hematoxylin and eosin staining was used to evaluate possible morphological changes in the aortic wall. The analysis did not reveal any alterations in the aorta morphology in the SHR-CRP and control SHR group (Fig. 2). Moreover, no inflammatory infiltration was visible in the aortas from either the SHR or SHR-CRP group.

### CRP increases oxidative stress but not inflammation biomarkers in the aorta

**Fig. 3:**
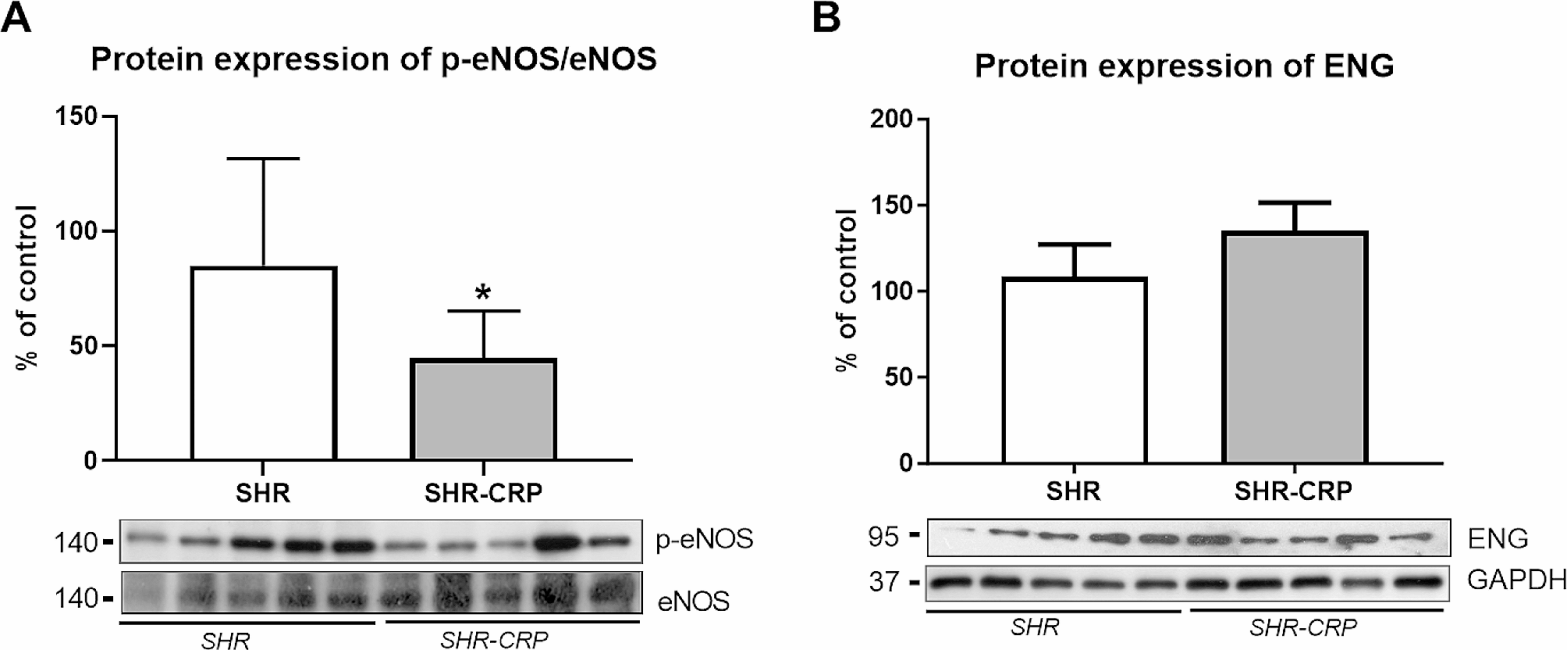
Expression of endothelial dysfunction markers in the aorta of SHR and SHR-CRP rats. Protein expression of p-eNOS/eNOS (**A**) and ENG (**B**) in total protein extracts from rat aortas. Top: densitometric analysis. Bottom: representative immunoblots. Data are presented as median with IQR and Mann-Whitney test. *p ≤ 0.05

**Fig. 4:**
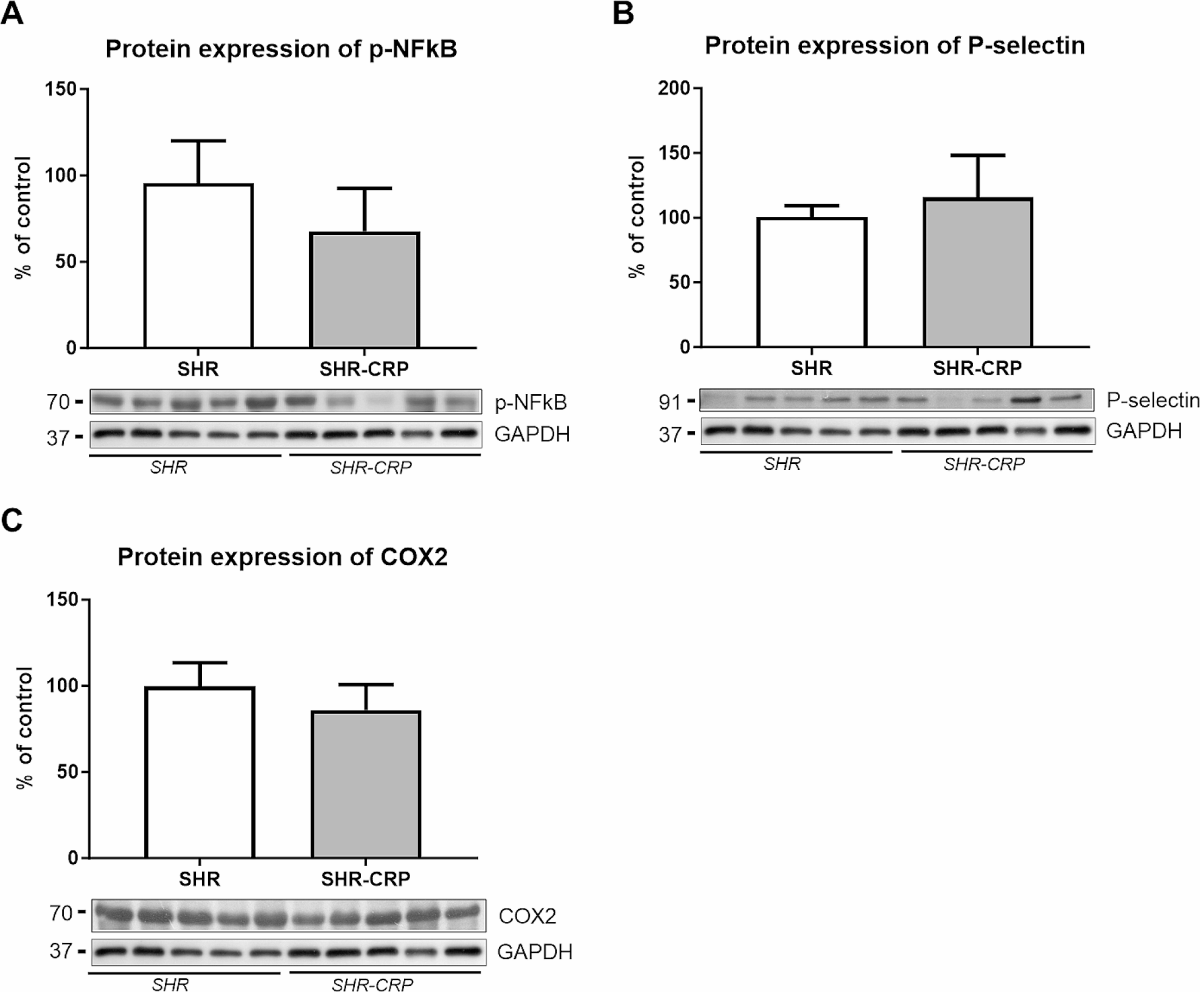
Expression of pro-inflammatory markers in the aorta of SHR and SHR-CRP rats. Expression of p-NFκB (**A**), P-selectin (**B**), and COX2 (**C**) in total protein extracts from rat aortas. Top: densitometric analysis. Bottom: representative immunoblots. Data are presented as median with IQR and Mann-Whitney test

**Fig. 5:**
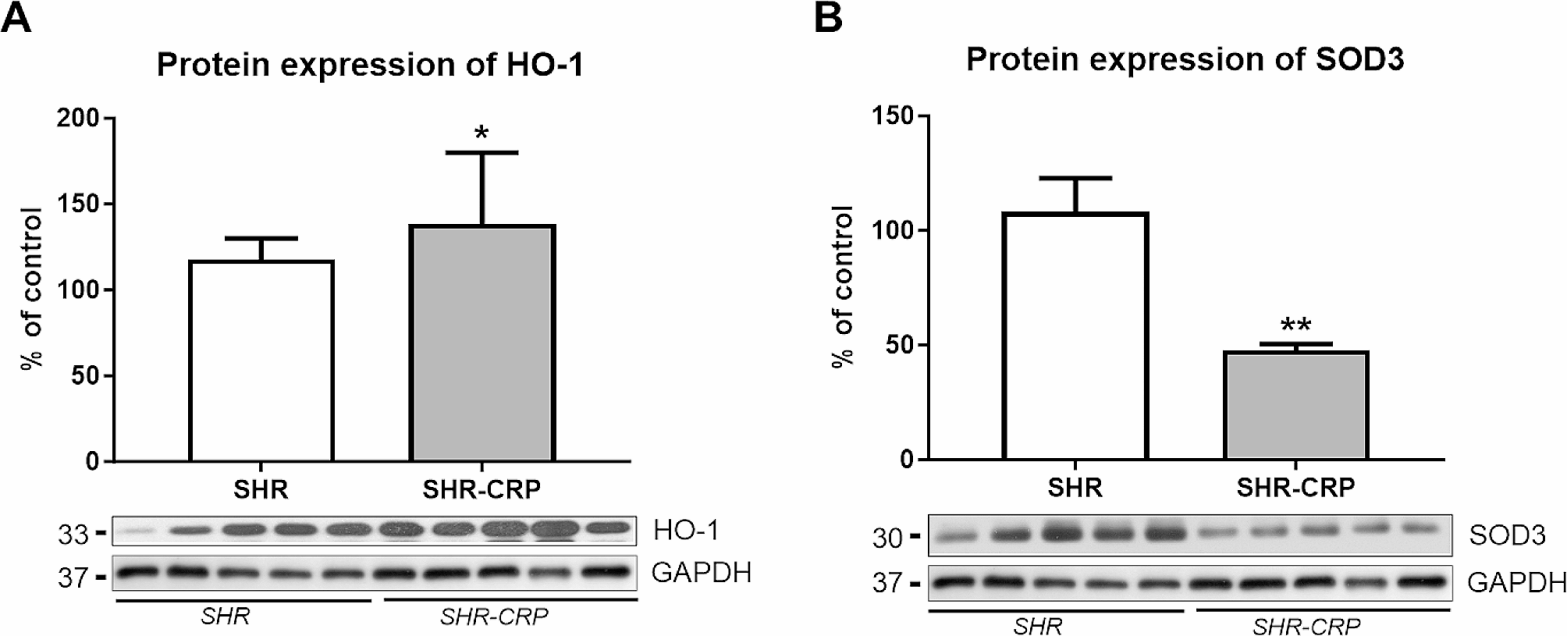
Expression of oxidative stress markers in the aorta of SHR and SHR-CRP rats. Protein expression of HO-1 (**A**) and SOD3 (**B**) in total protein extracts from rat aortas. Top: densitometric analysis. Bottom: representative immunoblots. Data are presented as median with IQR and Mann-Whitney test. *p ≤ 0.05, **p ≤ 0.01

Western blot analysis was used to reveal possible changes in the expression of markers related to endothelial dysfunction, inflammation, and oxidative stress in the aorta. The protein expression ratio of eNOS and its active phosphorylated form p-eNOS (Ser1177) revealed a significant decrease in the SHR-CRP group, while ENG expression was not significantly different between SHR and SHR-CRP rats (Fig. 3). The expression of inflammatory markers, p-NFkB, P-selectin, and COX2 was not significantly affected by the presence of CRP when compared to non-transgenic rats (Fig. 4). On the other hand, HO-1 expression was significantly increased, and SOD3 expression was significantly reduced in SHR-CRP rats compared to control non-transgenic animals (Fig. 5), indicating oxidative stress induction accompanied by redox imbalance.

## Discussion

CRP was shown to be the most studied biomarker of cardiovascular pathologies, particularly atherosclerosis and ischemic heart diseases [8]. However, there is still ongoing discussion about the possible role of CRP as a possible bystander or inducer of cardiovascular and metabolic alterations [13]. Recently, human CRP transgenic SHR-CRP rats were introduced to assess CRP’s role in the pathogenesis of metabolic syndrome [13]. Indeed, the presence of human CRP in these rats resulted in higher blood pressure, glucose levels, oxidative stress, and inflammation compared to non-transgenic animals [13]. However, until now, no study has been conducted to investigate the potential effects of CRP in the aorta.

Thus, we hypothesized that CRP affects the protein expression of biomarkers of endothelial dysfunction, inflammation, and oxidative stress in the aorta.

In this study, the presence of human CRP was associated with significantly decreased insulin-stimulated glycogenesis in skeletal muscle, increased insulin plasma levels, increased muscle and hepatic accumulation of TAG and decreased plasmatic cGMP concentrations, reduced adiponectin levels and increased monocyte chemoattractant protein-1 (MCP-1) levels in the blood, suggesting pro-inflammatory effects and presence of multiple features of metabolic syndrome in SHR-CRP animals as was partially shown previously [13]. Indeed, adiponectin is an important insulin-sensitizing adipokine expressed almost exclusively in adipose tissue and has also been reported to exert anti-diabetic, anti-atherosclerotic, anti-inflammatory, and cardioprotective actions. It has been shown that decreased levels are found in people with cardiovascular disease. Several studies showed that the weight and metabolic activity of visceral adipose tissue are important and may be related to reduced adiponectin concentrations [18]. However, we did not detect significant changes in these parameters, including the relative weight of epididymal adipose tissue, the relative weight of perirenal adipose tissue, and NEFA between SHR and SHR-CRP rats. Interestingly, the changes in adiponectin levels were associated with inflammation and oxidative stress, which was increased in the aorta in this study as well.

Indeed, adiponectin mediates several functions in vascular endothelium, e.g., production of NO via phosphorylation of eNOS at Ser1177 or attenuation of production of ROS [18, 19], suggesting that reduced levels of adiponectin might also be related to NO levels in aorta. Thus, we studied the expression of proteins related to the proper function of vascular endothelium. eNOS and its phosphorylated form p-eNOS (phosphorylated on Ser1177) are necessary for the proper production of NO and the regular function of the endothelium. It was demonstrated that CRP reduced the expression of eNOS (both gene and protein) in vitro in human aortic endothelial cells (HAECs), suggesting the direct participation of CRP in the development of endothelial dysfunction [6]. Furthermore, the reverse association of CRP and eNOS activity has been shown in rats after intraperitoneal administration of human CRP [5]. Western blot analysis demonstrated a significant decrease in the p-eNOS/eNOS protein expression, which is a typical hallmark of alteration of endothelial function. Interestingly, it was shown that ENG supports eNOS activity and expression, thus, it might be involved in endothelial protection [20], and its reduced expression was related to the aggravation of endothelial function [21]. However, western blot analysis conducted in the present study did not reveal significant changes in ENG expression between SHR-CRP and SHR, which suggests that ENG is not related to eNOS expression in these rats. It is of interest to mention that ENG expression in rat aorta has not been studied so far when compared to mice. Therefore, we cannot exclude the possibility of the different role of ENG in rat endothelial function.

The inflammation represents the hallmark of endothelial dysfunction, and it is characterized by the increased activity and expression of NFkB, which regulates the expression of cell adhesion molecules involved in the transmigration of leukocytes in early endothelial dysfunction [22]. Indeed, CRP was also shown to increase the expression of cell adhesion molecules in HAECs [6]. However, the presence of human CRP in SHR in this study did not affect NFkB, P-selectin, and COX2 expression in the aorta, suggesting that cell adhesion molecules and inflammation are not induced by the presence of CRP in the aorta of SHR-CRP rats.

The presence of CRP is also related to oxidative stress, as published previously [13]. Thus, we focused on oxidative stress-related biomarkers in rat aorta. HO-1 is a highly inducible vascular protective enzyme activated by various stimuli, including oxidative stress (e.g., LDL oxidation) and inflammation (e.g., TNF-α) [23]. Its expression represents the hallmark of oxidative stress, however, it may also be responsible for the protection [11]. We found significantly increased expression of HO-1 in rat aorta, suggesting the development of oxidative stress in SHR-CRP rats. In addition, the expression of extracellular SOD3 was reduced in SHR-CRP. It was proposed that SOD3, in cooperation with catalase, improves endothelial-dependent vasodilatation in various conditions by protecting the NO-mediated signaling, and restoration of SOD3 is necessary for the correction of vascular structure and function [24–26]. Thus, reduced expression of important superoxide scavenger SOD3 and increased HO-1 expression suggests oxidative stress induction in SHR-CRP rats.

Our study demonstrated, for the first time, that CRP is only partially able to affect biomarkers of endothelial dysfunction in SHR rats, which are genetically predisposed to the development of hypertension and metabolic syndrome. Thus, we suggest that CRP alone cannot fully induce the expression of endothelial dysfunction biomarkers, suggesting other risk factors of cardiovascular disorders are necessary to be involved in inducing endothelial dysfunction with CRP.

### Electronic supplementary material

Below is the link to the electronic supplementary material.

Supplementary Material 1

## Data Availability

All data are part of the submitted manuscript.
