# Supplementary material for: Transgenic human C-reactive protein affects oxidative stress but not inflammation biomarkers in the aorta of spontaneously hypertensive rats
Source: BMC Cardiovasc Disord. 2024 Apr 16;24:211. doi: 10.1186/s12872-024-03870-7 (PMC11020172; doi:10.1186/s12872-024-03870-7)

GAPDH

Molecular weight: 37kDa

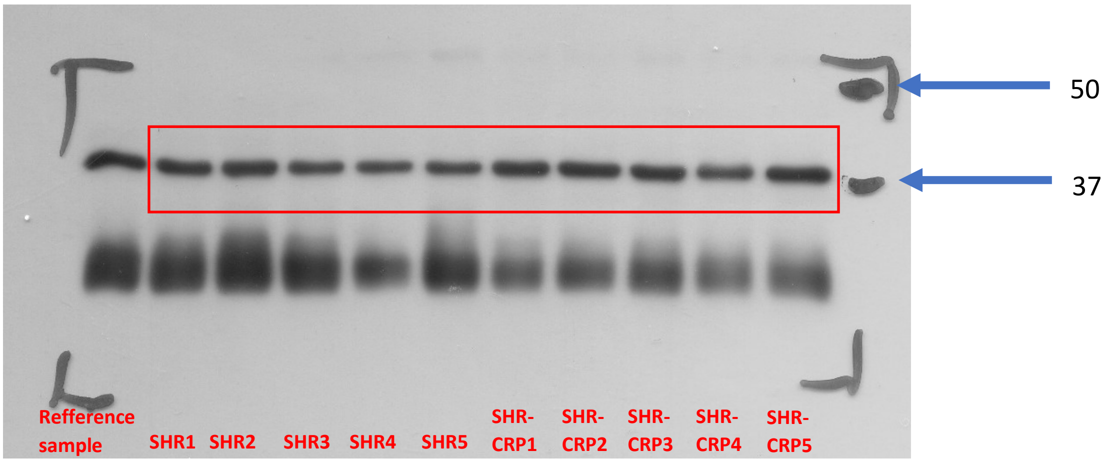

eNOS

Molecular weight: approximately 140kDa

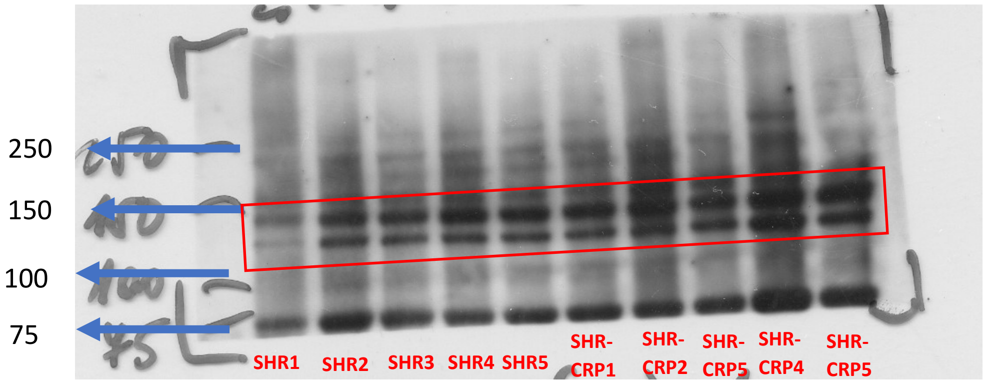

p-eNOS

Molecular weight: approximately 140kDa

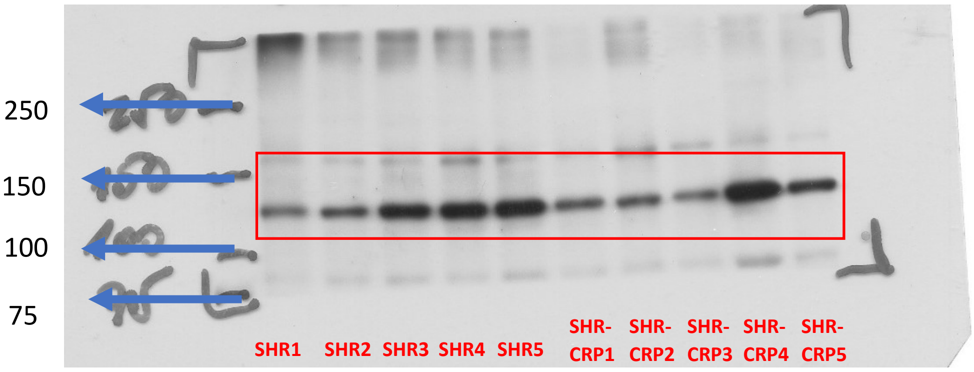

ENG

Molecular weight: approximately 95kDa

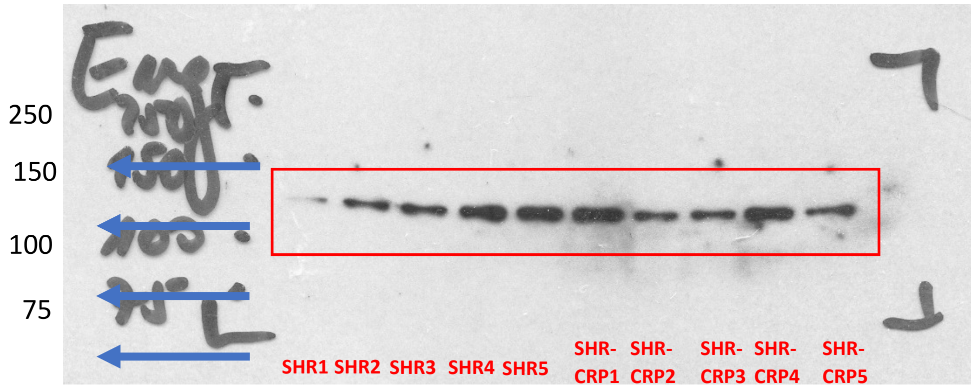

p-NFkB

Molecular weight: approximately 70kDa

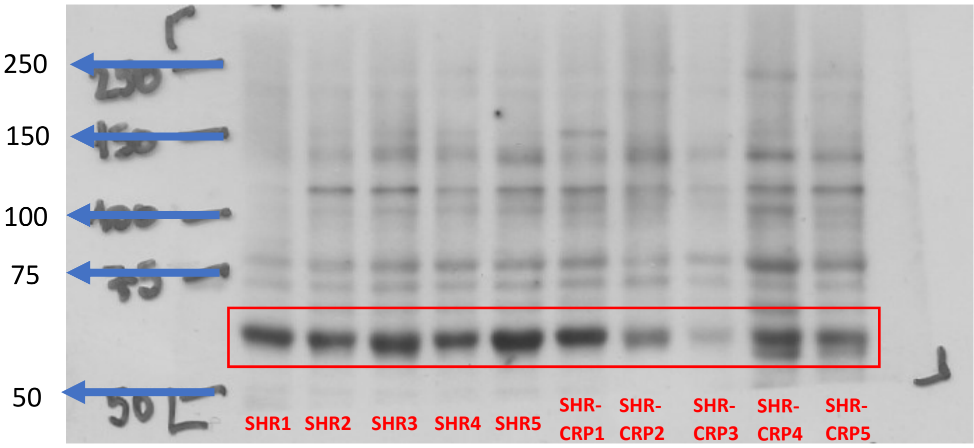

P-selectin

Molecular weight: approximately 91kDa

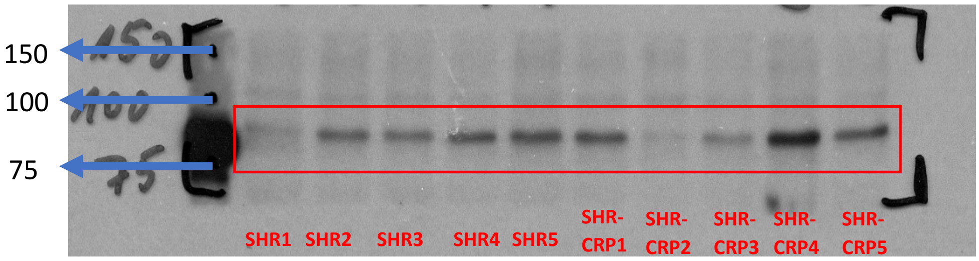

COX2

Molecular weight: approximately 70kDa

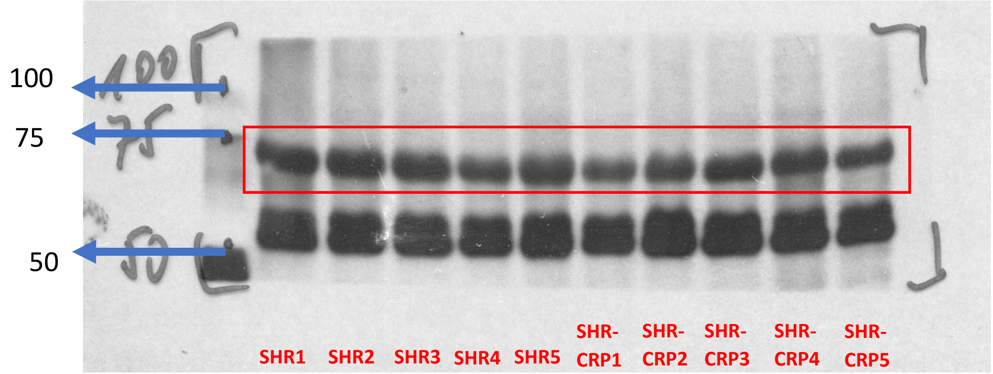

HO-1

Molecular weight: approximately 33kDa

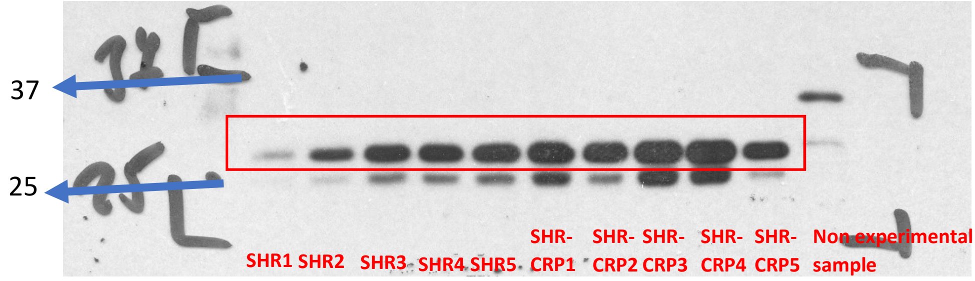

SOD3

Molecular weight: approximately 30kDa

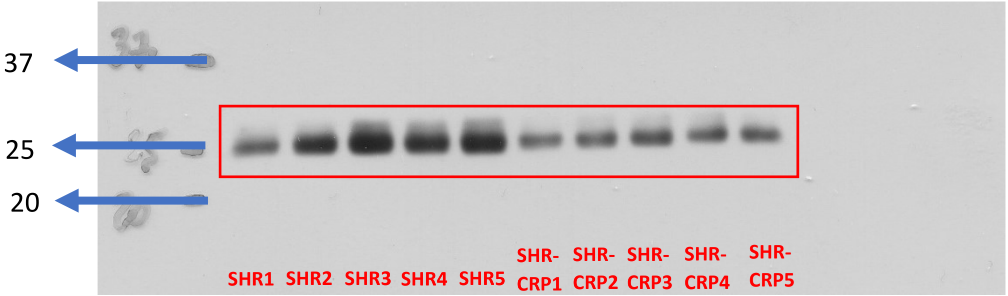

Supplement: Supplementary file 1 — Supplementary Material 1 [file 12872_2024_3870_MOESM1_ESM.pdf]
